# A case report of left atrial myxoma presenting with amnesia

**DOI:** 10.1186/s12872-021-02036-z

**Published:** 2021-05-01

**Authors:** Sadegh Shabab, Majid Erfanzadeh, Shamsa Ahmadian, Maryam Mahmoudabady, Naser Mazloum

**Affiliations:** 1 Department of Physiology, Faculty of Medicine, Mashhad University of Medical Sciences, Mashhad, Iran; 2 Department of Cardiology, Samen Hospital, Mashhad, Iran; 3 Applied Biomedical Research Center, Mashhad University of Medical Sciences, Mashhad, Iran

**Keywords:** Cardiac tumor, Myxoma, Cardiac surgery, Embolism, Case report

## Abstract

**Background:**

Primary cardiac tumors are rare, and approximately 90% of them are benign. Myxoma is the most common type of these tumors occurring in the left atrium in 75–85% of cases. The tumor can cause the left atrio-ventricular valve obstruction and embolization phenomenon.

Case presentation

We reported a case of 54-year-old man with complaints of dyspenea and amnesia. In our patient, transthoracic echocardiography revealed a mass of 28*63 mm attached to the upper intra-atrial septum, which was prolapsing through the mitral valve into the left ventricle during diastole, being indicative of the left atrial myxoma. On examination, he was alert and conversant, and no pathological abnormality was observed in the examination of cardiovascular, gastrointestinal, respiratory, hepatic, renal and nervous systems. After myxoma diagnosis, the tumor was removed under cardiac surgery and discharged under good conditions. In the telephone follow-up after discharge, the patient recovered and did not report the disease and surgery complications.

**Conclusions:**

Patients with cardiac myxoma are usually asymptomatic, but they may have manifestations related to the embolism phenomenon or intracardiac obstruction. Therefore, myxoma may represent an emergency. Surgery should be performed as soon as possible. If surgery is delayed, the patient may suffer from serious and irreversible complications, such as stroke and cardiac arrest.

## Background

Cardiac tumors are rare in medical cases. Primary cardiac tumors are included in approximately 5% of cardiac tumors, and their occurrence are estimated to be less than 0.03% [1]. Approximately 90% of primary cardiac tumors are benign. The most common type of benign tumors of the heart is myxoma and occurs in the left atrium (LA) in 75–85% of cases [2, 3]. They generally occur more in females and after the third decade of life [4]. The tumor can cause the atrio-ventricular valve obstruction as well as the embolization phenomenon by throwing clots into systemic and pulmonary circulation [1, 5]. Systemic embolization occurs in 30–40% of cases of atrial myxoma [6]. Neurological manifestation and stroke are the most serious charges, and arrhythmia, heart failure and pericardial effusion are cardiac manifestations [1].

The manifestations of myxoma depend on its size, location, and mobility [7]. Patients may experience symptoms, such as dyspnea, syncope, angina, vertigo, fatigue, cough, and fever; however, they can be asymptomatic [8]. Transthoracic echocardiography (TTE) is the common method to diagnose myxoma [9]. Currently, surgical removal of the tumor mass is the best treatment, and there is no effective medical treatment confiscating the tumor growth [1]. Here, we present a 54-year-old patient with the left atrium myxoma, who referred to a heart clinic on an outpatient basis.

## Case presentation

A 54-year-old man referred to our heart clinic department with complaints of dyspnea for 3 days. In the evaluation of the patient, he stated the history of COVID-19 disease 2 months ago and amnesia for approximately 6 months ago, so that he sometimes lost his way home, he also did not remember the names of people close to him, such as his wife and children. He does not report other diseases and intervention. The patient also did not report psychosocial abnormality, drug use, smoking, opium and alcohol as well as family history of the disease.

On examination, he was alert and conversant. His vital signs were stable with the blood pressure of 118/79 mmHg. No pathological abnormality was observed in the examination of cardiovascular, gastrointestinal, respiratory, hepatic, renal and thyroid systems. The patient currently had no COVID-19 signs and symptoms. No abnormality was demonstrated in the auscultation of the heart sound. Further evaluation of central and peripheral nervous systems did not report symptoms, such as diplopia, headache, dizziness, nausea, and numbness.

The complete blood count, lipid profile, and renal and liver function tests were normal. No abnormalities were observed in white blood cells and lymphocyte counts. Erythrocyte sedimentation rate (ESR) was increased to 26 mm/h, and C-reactive protein (CRP) was positive. In blood biochemistry studies, serum levels of sodium, potassium, calcium, phosphorus, and magnesium ions were in the normal range.

**Fig. 1:**
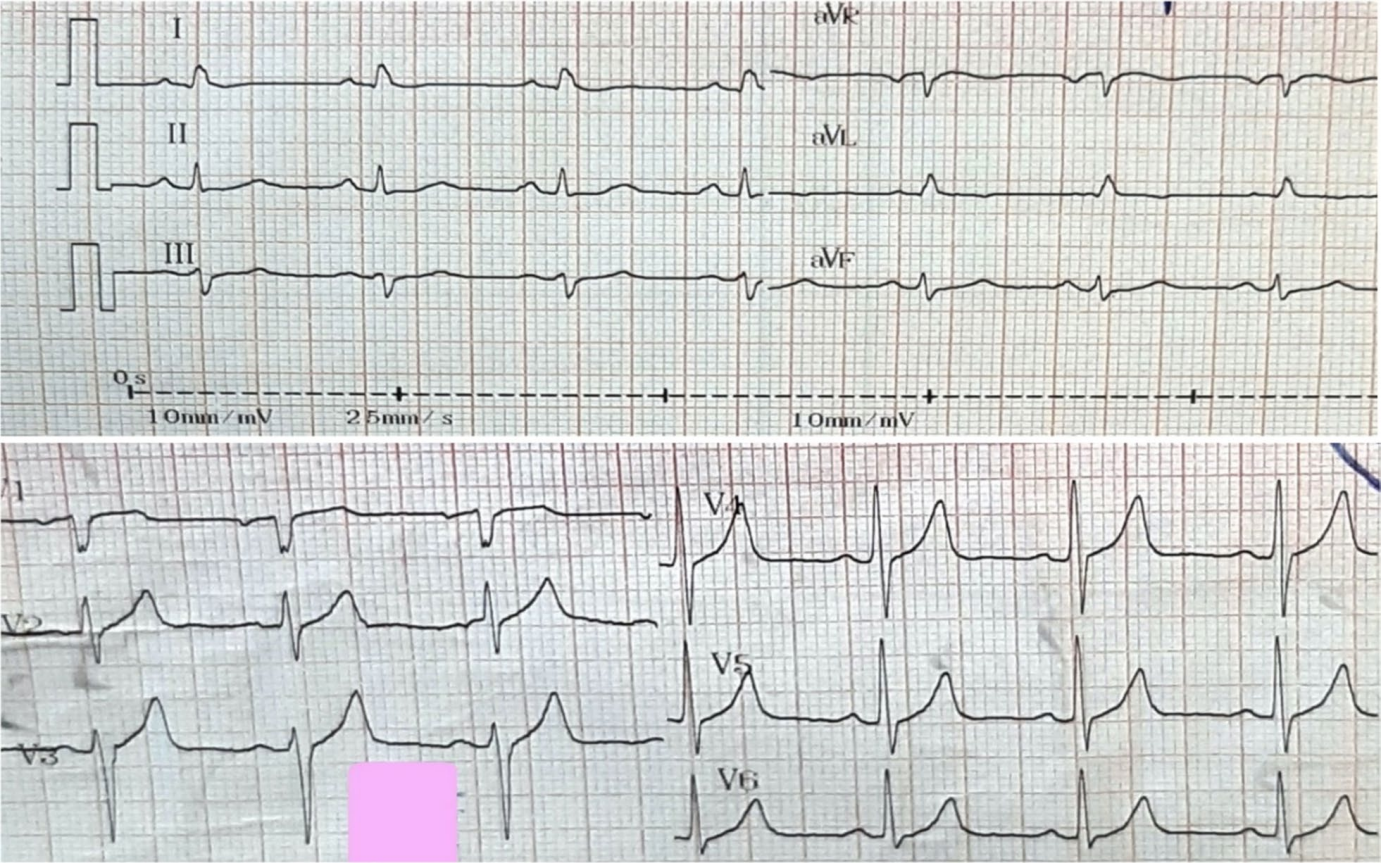
ECG shows no pathological changes in limb and pericardial leads

**Fig. 2:**
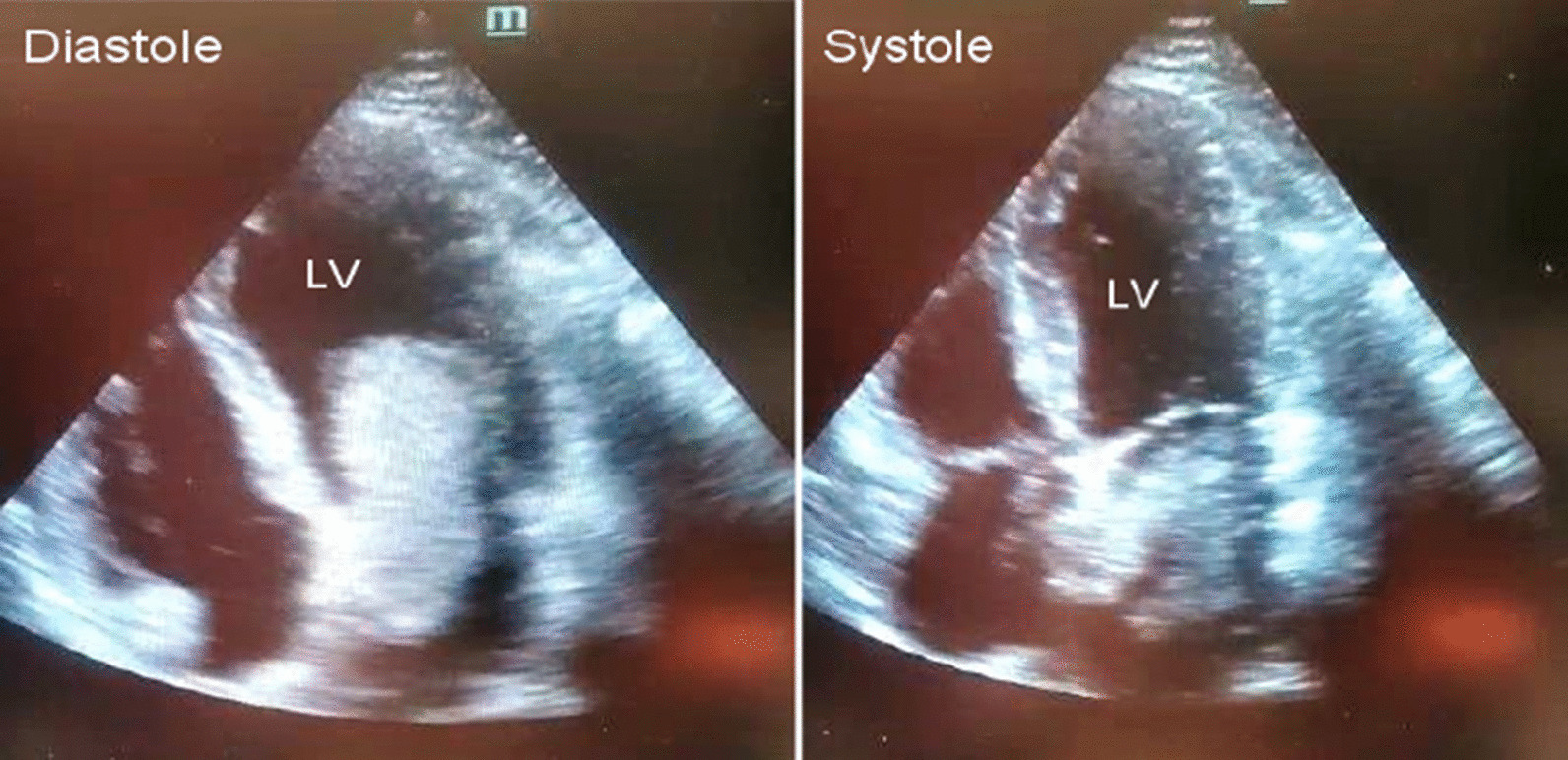
Transthoracic echocardiography shows the left atrial myxoma prolapsing through the mitral valve into the left ventricle during diastole and trapping in the left atrium during systole

As Fig. 1 shows, no pathological changes were observed in the electrocardiography (ECG) taken from the patient. As Fig. 2 displays, in TTE, a mass of 28*63 mm attached to the upper intra-atrial septum prolapsing through the mitral valve into the left ventricle during diastole was revealed, indicating the left atrial myxoma. Due to early TTE and tumor diagnosis, other diagnoses like acute coronary syndrome were evaluated and rejected.

Since we had limited medical equipment, and due to the patient’s emergency and life-threatening conditions, we were satisfied with the result of TTE, and no additional studies, such as CT scan and MRI, were conducted.

The patient was immediately admitted to the cardiac intensive-care unit. In cardiac and respiratory monitoring, the patient had a normal sinus rhythm without dysrhythmia, and the arterial oxygen saturation (Spo2) level was 98%. In the serial examination of vital signs, BP was 120/80, and HR and the respiratory rate were 75/min and 17/min, respectively. The patient did not have a fever. He was kept on anticoagulants, antiplatelets and nitrate agents.

In addition, in the complementary TTE examination, ejection fraction (EF) was 55%, and no abnormalities were observed in the valves, thickness and movements of the walls of the heart cavities. Pericardial effusion and increased pulmonary artery pressure were also not observed.

**Fig. 3:**
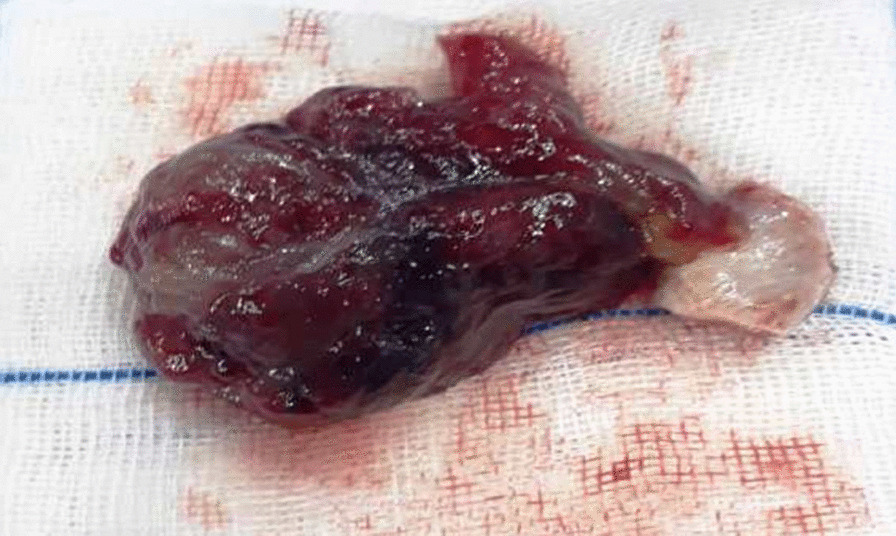
The soft mulberry-shaped mass harvested from the left atrium

The patient underwent coronary angiography immediately. Non-significant lesions were observed. After angiography, under general anesthesia, open-heart surgery was performed. After thoracotomy and cardiopulmonary bypass, the heart was arrested with cold blood cardioplegia. As Fig. 3 depicts, to have access to the mass, by cutting into the left atrium of the heart wall and reach, a 28*63 mm soft mulberry-shaped mass was harvested. The left atrium was closed in layers and weaned off from the cardiopulmonary bypass without a pressor or inotropes. His heart spontaneously returned to normal sinus rhythm and did not require defibrillation. The chest was closed in layers.

The patient was transferred to the recovery room. After the patient’s vital signs in recovery were stable, he was transferred to the intensive care unit of cardiac surgery and monitored. He underwent the intensive care of cardiac surgery for two days and then was transferred to the Cardiac Care Department and was discharged in good condition after three days. After performing angiography and surgery, the patient was satisfied with the treatment process and did not report any particular complication regarding the treatment protocol.

**Fig. 4:**
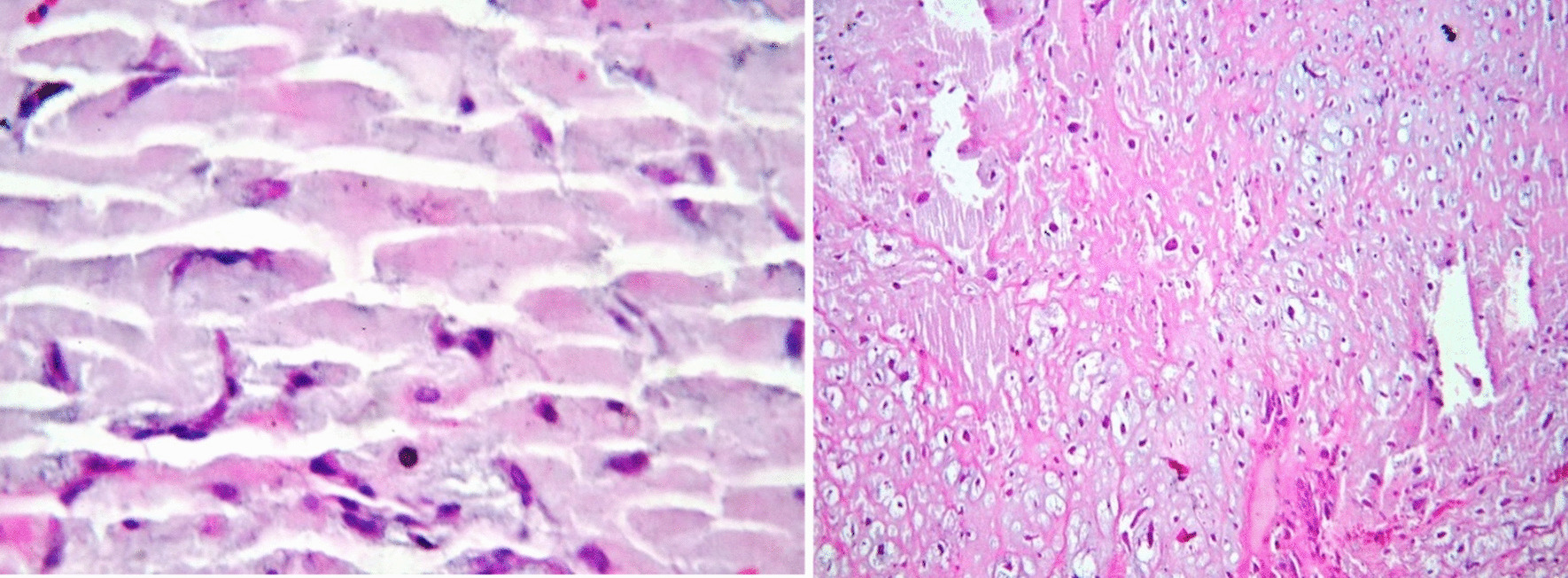
A histological examination showing free neoplastic sections with extensive myxoid stroma containing spindle and star-shaped cells, creating tricular and quasi-glandular shapes

For pathology studies, the sample was sent to the Pathology Department. In the pathology report, a specimen with dimensions of 4 × 2.5 × 1.5 in cream to brown color with soft consistency was reported. On microscopic examination of the tissue, free neoplastic sections with extensive myxoid and relatively vascular stroma containing spindle and star-shaped cells without atypia and significant mitosis were visible, creating tricular and quasi-glandular shapes. As Fig. 4 shows, the pathological study of the mass confirmed the diagnosis of LA myxoma.

At the patient's return visit after 10 days, the patient's condition was assessed as stable. In the telephone follow-up for 3 months after discharge, the patient recovered and did not report the disease and surgery complications. Furthermore, according to the patient's family, after surgery, forgetfulness occurs less often in the patient.
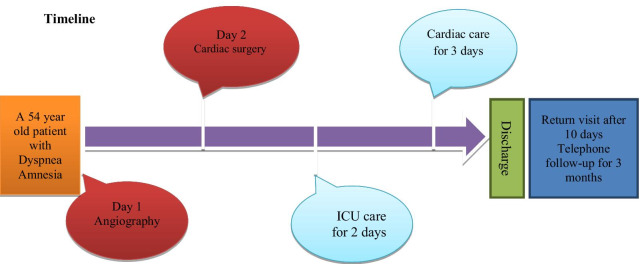


## Discussion and conclusion

Primary cardiac tumors are rare and approximately 90% of them are benign. Myxoma is the most common type of the benign tumor of the heart occurring in the left atrium in 75–85% of cases [2, 3].

We reported a case of 54-year-old man with complaints of dyspenea and amnesia. In our patient, TTE revealed a mass of 28*63 mm attached to the upper intra-atrial septum. TTE is preferred to diagnose cardiac myxoma, but transesophageal echocardiography (TEE) provides a better description of the tumor location and characteristics. Thus, TEE is preferred over TTE to evaluate the left atrial myxoma [1]. Since we had limited medical equipment, due to the patient’s emergency and life-threatening conditions, we were satisfied with the result of TTE, and no additional studies, such as CT scan and MRI, were performed.

Heart sound auscultation may contribute to myxoma diagnosis, although heart sound abnormalities may be absent in 36% of myxoma cases [10]. No abnormality was demonstrated in the auscultation of the heart sound in our patient.

The patients are usually asymptomatic, but they may have manifestations related to embolism and/or intracardiac obstruction [11]. Embolic events occur in approximately 30–40% of patients with cardiac myxoma. It often occurs in the brain owing to fragments of the myxoma itself or surface embolism. In addition, the embolization phenomenon may occur in the spleen, kidneys, aortic bifurcation, and lower extremities [12]. Transient ischemic attack (TIA) is the most common neurological presentation occurring in multiple sites with psychiatric symptoms. Cerebral ischemia caused by atrial myxoma accounts for only 0.5% of all strokes [1]. No abnormality symptom of central and peripheral nervous systems, such as diplopia, headache, dizziness, nausea, and numbness, were observed in our patient.

The patients may experience symptoms, such as dyspnea, syncope, angina, vertigo, fatigue, cough, and fever [8]. Our patient complained of dyspnea for 3 days. Additionally he suffered from amnesia for approximately 6 months, so that he sometimes lost his way home, he also did not remember the names of people close to him, such as his wife and children. According to the patient's age and no family history of Alzheimer's, the possibility of throwing microembolism into the central nervous system and causing symptoms such as forgetfulness is expected. However, amnesia can be a symptom of other diseases, such as schizophrenia and dementia. Neurodegenerative disorders, such as Alzheimer’s, Parkinson and Huntington disease, should also be regarded as other causes of amnesia [13].

The cases of the left atrial myxoma often suffer from hemolytic anemia and thrombocytopenia caused by the mobile intraluminal tumor and the mechanical destruction of blood flow [1]. In our case, the tumor was prolapsing through the mitral valve into the left ventricle during diastole, but neither this nor that symptom was observed in our patient.

 Because the patient was educated, the surgeon explained the conditions of the disease and possible complications to him. The patient also received additional training on diagnostic and treatment protocols. While accepting the condition, the patient consciously requested surgery and removal of the tumor. After performing angiography and surgery, the patient was satisfied with the treatment process and did not report any particular complication regarding the treatment protocol.

Since myxoma represents an emergency, surgery should be performed as soon as possible after diagnosis is established. Embolism and valve obstruction are acute complications due to surgery postponement [1]. Therefore, was surgery performed as soon as possible for our patient. One of the limitations of our study was the lack of additional studies and diagnoses, such as CT scan and MRI, which are recommended in similar studies.

Given that many of these patients are asymptomatic or have nonspecific symptoms, it appears that by taking a detailed history and conducting clinical examinations, as well as considering additional diagnostic parameters, such as TTE, CT scan and MRI, myxoma can be diagnosed and treated early. It appears that due to the availability and cost-effective of TTE, it should be used for early detection of the cardiac myxoma. After diagnosis, surgery should be performed as soon as possible, otherwise the patient may suffer from serious and irreversible complications, such as stroke and cardiac arrest. After surgery, long-term follow-up is necessary.

## Data Availability

The datasets used and/or analysed during the current study are available from the corresponding author on reasonable request.

## Abbreviations

LA: Left atrium
TTE: Transthoracic echocardiography
ESR: Erythrocyte sedimentation rate
CRP: C-reactive protein
ECG: Electrocardiography
Spo2: Oxygen saturation
EF: Ejection fraction
TEE: Transesophageal echocardiography
TIA: Transient ischemic attack
